# Is early silcrete heat treatment a new behavioural proxy in the Middle Stone Age?

**DOI:** 10.1371/journal.pone.0204705

**Published:** 2018-10-01

**Authors:** Regine E. Stolarczyk, Patrick Schmidt

**Affiliations:** 1 Department of Prehistory and Quaternary Ecology, Eberhard Karls University of Tübingen, Tübingen, Baden-Württemberg, Germany; 2 Research Center ‘The Role of Culture in Early Expansions of Humans’, Heidelberg Academy of Sciences and Humanities housed at the University of Tübingen, Baden-Württemberg, Germany; 3 Department of Geosciences—Applied Mineralogy, Eberhard Karls University of Tübingen, Tübingen, Baden-Württemberg, Germany; University at Buffalo - The State University of New York, UNITED STATES

## Abstract

The South African Middle Stone Age (MSA) has in recent years become increasingly important for our understanding of the emergence of ‘modern human behaviours’. Several key innovations appeared in this context for the first time, significantly pre-dating their re-invention in the European Upper Palaeolithic. One of these innovations was heat treatment of stone to improve its quality for the production of stone tools. Heat treatment may even be the oldest well-documented technique used to intentionally alter the properties of materials in general. It is commonly thought of as requiring the skilled use of fire, a high degree of planning depth and complex cognitive abilities. However, to work on these fundamental concepts we need to analyse the techniques and procedures used to heat-treat and we need to understand what they imply. In this paper, we present a direct and expedient comparison between the technical complexities of four alternative heat treatment procedures by coding the behaviours required for their set-up in so-called cognigrams, a relatively new method for understanding complexity based on the problem-solution distance. Our results show that although the techniques significantly differ in complexity, the techniques used in the MSA fall within the range of complexities known from other MSA techniques. Heat treatment in above-ground fires, as it was practised during this period in South Africa, was even one of the most complex techniques at the time of its invention. Early heat treatment can therefore be considered an important behavioural proxy that may shed light on the behaviour and socioeconomic structure of past groups. The implications of this are highlighted by the ongoing debate about ‘modernity’, ‘behavioural flexibility’ and ‘complex cognition’ of early anatomically modern humans in Africa.

## Introduction

The African Middle Stone Age (MSA) is a timespan between 300 and 30 ka. Its study yielded important results for our understanding of what some authors have described as ‘modern behaviours’ [1], others as ‘behavioural variability’ [2] and still others as the advent of ‘complex cognition’ [3]. The MSA saw the first appearance of *Homo sapiens* at c. 300 ka in North Africa [4] and its expansion all over Africa. The dispersion of anatomically modern humans into southern Africa [5] roughly falls in the second half of the MSA. Several new traits appearing in the later part of the MSA have been interpreted to be key innovations that define the unique behaviour of early anatomically modern humans. Such traits include the use of marine resources [6], bone tools [7, 8], symbolic behaviours [9–12], adhesives and compound tools [13–15] and complementary tool sets such as bow and arrow [16]. A new possible member on this list of traits is controlled heat treatment of silcrete for stone tool production [3, 17, 18]. Heat treatment is the intentional modification of a stone’s properties by fire. It was used in various contexts all over the world to either facilitate stone knapping (see for example: [19–23]) or to produce more efficient stone tools [24, 25]. However, MSA heat treatment is not considered a potential proxy of sophisticated hominin behaviour because of the technological advantage it procures for tool production, but because of the cognitive investment necessary for its execution. To interpret early heat treatment, the cognitive processes associated with it must thus be analysed. One possible measure of the investment in cognitive effort and strategic thinking necessary for archaeological processes is to describe their complexity [26]. The concept of complexity has been used to describe complete cultural systems (see for example: [27, 28]) but it has also been used on a single process scale, where it is often defined as the number of steps needed to complete a procedure [29]. Unfortunately, there is no unanimously accepted way to quantify complexity [30]. A promising approach is the one using so-called ‘cognigrams’ [31, 32]. The method proposes an à priori unbiased measurement of the complexity of technological processes by quantifying the problem-solution distance (being defined as all objects, actions, effects and thoughts implicated in the process). Such an analysis can potentially be used to interpret prehistoric heat treatment if all steps and objects involved in the heating technique are known or can be inferred from the available data. Thus the techniques used for heat treatment must be investigated and the resulting technical interpretation must be analysed before attempting to draw conclusions on the implications of the process for the cultural evolution of early *Homo sapiens* in southern Africa.

Few direct archaeological data are available on the technique used for heat treatment in the MSA. Schmidt et al., [33] recently found that silcrete was heated in direct contact with the embers of open-air fires, notwithstanding previous hypotheses [3, 17, 18, 34] on the use of underground heating in the southern African MSA [35]. To date, above-ground heat treatment in a fire has been reported from several sites in South Africa’s west and south coast regions (see for example: [33, 36–38]) and no archaeological data that would indicate underground heating has so far been reported in the MSA. Thus, it appears that interpreting early heat treatment requires understanding the heating processes associated with open-air fires. However, to evaluate the implications of heat treatment for the MSA, we attempt to view the heating technique used for it in the broader context of heating techniques known from other contexts. In this paper we propose a comparative study of the most common archaeologically documented technical processes, used to heat stone at different periods and in different parts of the world, to interpret early silcrete heat treatment in the broader context of MSA innovations.

## Methods and materials

### Archaeological evidence of techniques used for heat treatment

Although numerous archaeological contexts where heat treatment was used to manufacture stone tools were found on all continents [17, 19–21, 23, 39–42], there are few direct archaeological data that would actually document the techniques used for heat treatment. There are also numerous experimental approaches that do not rely on archaeological data (see for example: [17, 18, 43, 44]) but we chose to only analyse techniques for which at least some direct archaeological data, documenting the procedure, is available. Based on this available archaeological literature, two groups of techniques can be distinguished: (I) heat treatment using the above-ground fire and (II) underground heating. Groups (I) and (II) can be divided in two sub-groups each: above-ground heating was described as (i) heating directly in the ash- and embers-cone of a fire and (ii) at the periphery of the fire [33]; underground heating was described as (i) a sand-bath [45] and (ii) an earth-oven like fire-pit [46]. To date, these four variations, appear to be the only archaeologically documented heating techniques. Our comparative analysis is based on the interpretation of these four techniques.

#### Above-ground heating in a fire or with the ‘pile of embers’ technique

**Fig 1:**
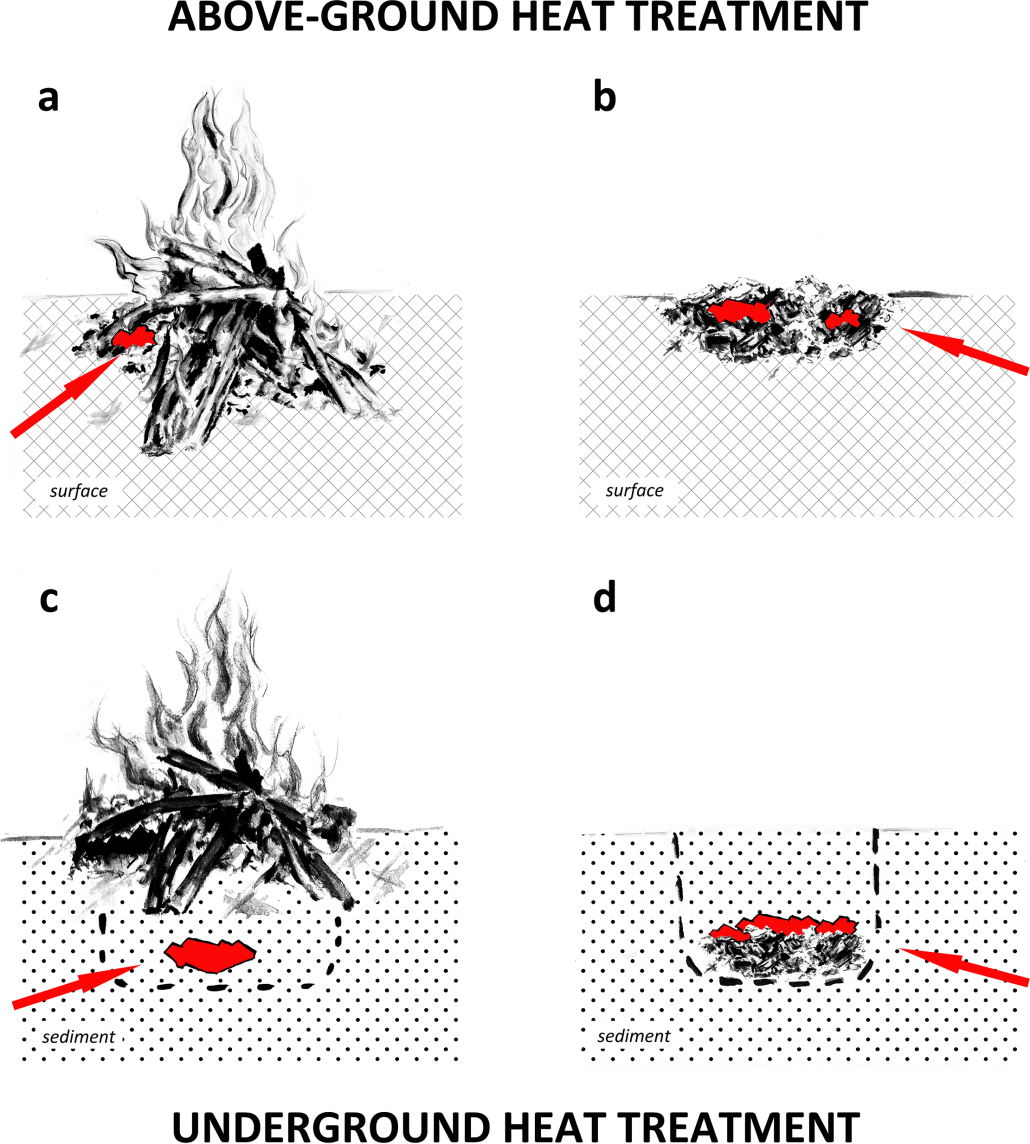
Heating techniques compared by this study. a: Above-ground heating in a fire; b: Above-ground heating with the ‘pile of embers’ technique; c: Underground heating in a sand-bath; d: Underground heating in an earth-oven like fire-pit.

In a recent study on heat-treated silcrete from the South African MSA, Schmidt et al. [33] found two above-ground heating techniques. An organic residue on heat-treated silcrete artefacts from Diepkloof Rock Shelter [47] indicated that the stones were in contact with embers during the treatment. The presence of heat-induced fracturing, resulting from explosive events during the treatment, further indicated fast heating rates such as the ones produced if stone is heated in above-ground fires. Two potential heating procedures were proposed as an interpretation of these results: (I) heat treatment directly in the ash- and embers-cone of an open-air fire and (II) heat treatment in embers at the periphery of a fire. Based on these assumptions the simplest scenario for (I) is: [1] stone is placed onto the ash- and embers-cone of a campfire. The process ends when the stone is (more or less slowly) pulled out from the fire. The simplest scenario for (II) is: [1] embers are separated from the campfire and placed at its periphery; [2] stone is placed in the separated embers. The process ends when the embers cool down to a temperature at which the stone can be touched again. In both cases an existing campfire can be used. Fig 1A and 1B are schematic representations of these two above-ground heating techniques. Both techniques are well suited for coarser-grained rocks like South African silcrete, the chemistry and structure of which do not require low temperatures and slow heating rates [48, 49]. However, finer grained silica rocks like flint and chert cannot be heated in this way because the fast heating rates and high temperatures would result in the material shattering into unusable pieces [50, 51]. For coarse grained rocks that withstand such conditions above-ground heating has the advantage of being relatively less resource, time and labour intensive as compared to underground heating (compare [33, 52]).

#### Underground heating in a sand-bath

The sand-bath heating technique consists of burying stone in sediment beneath a fire. The stone is indirectly heat-treated by the heat transfer in the sand, resulting in relatively slow heating rates and low temperatures. Although sand-bath heating is widely accepted by archaeologists and experimenters [17, 18, 43, 53, 54], to our knowledge there are only two detailed descriptions of archaeological structures that can be interpreted as sand-baths: Clark and Khana’s [55] description of a pit with reddened walls found in central India and McDonald and Rich’s [45] description of a Holocene heat treatment pit in eastern Australia. Of these two, only the latter report provides sufficient detail to infer a potential heating procedure. McDonald and Rich describe a pit within hardened clay that was filled with silt. Artefacts were discovered below a lump of charcoal. The authors interpret the structure as a partially, but not completely destroyed heat treatment pit. Unfortunately, the description lacks a detailed section-drawing, making it not entirely clear whether the artefacts were separated from the charcoal by a consistent layer of sediment or whether they were in contact with the lower part of the charcoal. If the former scenario was indeed the case, the structure may have been the leftovers of sand-bath heating; in the latter case, it may have resulted from heat treatment at the base of an open-air fire. Based on the author’s interpretation and the available recent and sub-recent ethnographic data on sand-bath heating (see for example: [56–58]), the simplest scenario is: [1] a hole is dug; [2] stone is put in the hole and covered by a consistent layer of sediment; [3] a fire is lit on this sediment layer. The process ends when the fire burns out and the sand cooled down to a temperature at which the stone can be excavated. It is important to note that a dedicated fire must be lit on the surface of the sand-bath. If the structure is to be setup with precision (position of the stone under the fire, thickness of the sediment cover) the stone cannot be buried under an already burning camp-fire. Fig 1C is a schematic representation of such a sand-bath. Sand-bath heating has proven successful to heat-treat fine-grained silica rocks like flint and chert [43, 54] that would not withstand faster heating because of their chemical composition and structure [51, 59]. However, the sand-bath described by McDonald and Rich’s [45] was used to heat-treat *a priori* coarser-grained Australian silcrete. Thus, attempts to interpret the instigator’s choice of this specific technique for this material must await a detailed study on the structure and chemistry of the type of silcrete that was heated.

#### Underground heating in an earth-oven like fire-pit

The perhaps most detailed description of a structure used for heat treatment was made by Shippee [46] who interpreted an undated feature found in North America as a fire-pit for heat treatment of flint. He described a ∼45 cm-deep pit containing an infill of flint and ashes. The pit contained at its base a bed of ashes. Flint cores and flakes were placed on top of the ashes. The pit was backfilled with sediment and limestone boulders on top of the flint. Based on this description the simplest scenario is: [1] a hole is dug; [2] a fire is lit in the hole; [3] when the fire produced sufficient embers the stones are placed on the enbers; [4] the hole is backfilled. The process ends when the structure cooled down to a temperature at which it can be excavated and the heat-treated stone is extracted. As for the previous technique, it is important to note that underground heating in such a fire-pit is only possible if a dedicated fire is lit in the pit. Fig 1D is a schematic representation of such an earth-oven. A similar heating technique was successfully used to heat-treat fine grained silica rocks like flint and chert [44]. Due to the relatively low temperature and slow heating rates, produced by the embers glowing in reducing conditions in the sediment, an earth-oven is well adapted to heat-treat flint and chert that require such conditions to avoid unwanted fracturing [50]. The downside of underground heating, in an earth-oven or a sand-bath, is that they require relatively high investment in resources, labour and time [52].

### Analysis of the heating techniques using the cognigram method

**Fig 2:**
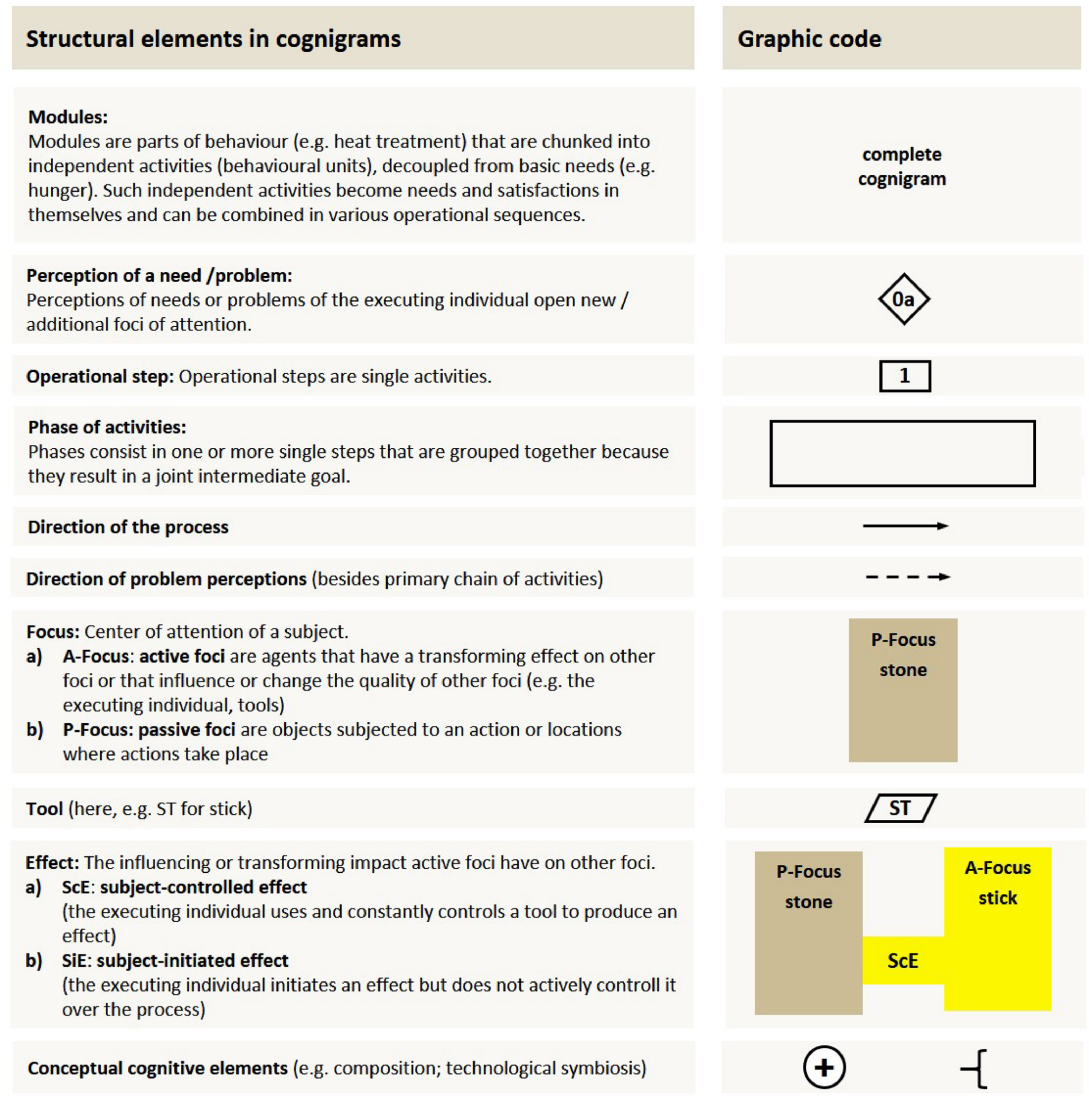
Definition and graphic code of structural elements in cognigrams. These symbols are: perception of need/problem; operational step; phase of activities; direction of process and problem perceptions; foci; tools; effects and other conceptual cognitive components (based on [31, 32, 60, 63]).

The method we use to compare the technological, and to some extent cognitive, complexities of these four alternative heating techniques was first developed by Haidle [31] and refined by Haidle [32]. Cognigrams are schematic representations of actualistic and materialistic interpretations of past behaviour inferred from the available archaeological and ethnographic data. They are based on the analysis of the *chaîne opératoire* [60] that is commonly used to interpret tool manufacturing in archaeological contexts (see for example: [61, 62]). In contrast to the chaîne opératoire approach however, which has a strong emphasis on the work piece, Haidle’s method shifts the focus to an activity-based perspective, which takes into consideration the whole behaviour. This approach attempts to systematically visualise the problem-solution sequence of a process (i.e. how all activities were organised, what objects were involved and the knowledge of actions and concepts that the instigator was required to understand in order to successfully achieve the intended task) in cognigrams. Such cognigrams are graphical representations of what Köhler [63] called the problem-solution distance: This problem-solution distance does not only include the length of the operational sequence, but also the underlying thought process and the nature and number of items and problems occurring during the process (the executing individual, tools, objects, locations or secondary goals). Furthermore, cognigrams also examine the potential interactions between foci and conceptual cognitive elements like technological symbiosis and composition. There are two basic requirements for encoding processes in cognigrams: (i) to choose the simplest perceivable technological pathway when interpreting the process [31, 32]. For example, if a camp-fire that can theoretically be used for heat treatment already exists at the site, it should be assumed that the executing individual used the existing camp-fire rather than lighting a supplementary fire. Secondly, (ii) equivalent starting and end points should be identified to provide comparability of problem-solution sequences. Therefore, every cognigram starts with the basic need and ends with the final accomplishment (i.e. the satisfaction of this need). Following this process, cognigrams of different technological processes or behaviours can be compared. A cognigram consists of codes for three main levels of analysis: (I) the nature and number of so-called foci of attention, (II) the sequence of actions executed during the process and (III) the effects different foci have on each other. Each of these categories is represented by distinct symbols (Fig 2). Additional elements like composition or technological symbiosis can be coded in cognigrams and are also depicted graphically [31, 32].

#### Foci in a cognigram

The first level of graphic representation in a cognigrams is a set of foci. They are depicted as vertical coloured bars (Fig 2). A focus is defined as the centre of attention of a subject. This includes the executing individual itself, all tools, objects and locations implicated in a process. Foci can be active or passive. Active foci can be described as agents that have a transforming effect on other foci or that influence or change the quality of other foci. The most common example of active foci is the executing subject itself and tools he/she uses. Passive foci are objects subjected to an action or locations where actions take place (Fig 2) [31, 32].

#### The sequence of actions in a cognigram

The *chaîne opératoire* of a technological process is subdivided into distinct operational steps and phases. Steps are defined as single activities. They are depicted as squares and labelled with sequential Arabic numerals (Fig 2). Phases consist of one or more single steps that are grouped together because they result in a joint intermediate goal. An example of a phase is lighting firewood with a burning branch picked up from a campfire. Such a phase cannot be interrupted without repeating the whole sequence of steps within the phase. In the above example this means that if the sequence of actions is interrupted in the middle, i.e. the burning branch is dropped after picking it up, the whole phase must be started over again. Phases are graphically represented as dashed rectangles, horizontally spreading over all involved foci, and labelled with Roman numerals (Fig 2) [31, 32].

#### Effects in a cognigram

The third level of graphic representation is the effect of a focus onto another focus. Such an effect is defined as the influencing or transforming impact active foci other than the executing individual (i.e. tools) have on other foci and illustrates interactions/connections between foci. The executing individual uses tools and their specific qualities as causal agens to change the form, condition or position of a target [31, 32]. They are illustrated by coloured horizontal bars between foci (Fig 2). We adapted Haidle’s method concerning effects in two ways: (i) we distinguished different types of effects depending on the level and nature of control exercised by the executing individual. Two types of effect are of significance for this study: the subject-controlled effect (ScE) and the subject-initiated effect (SiE). In the case of a ScE the executing individual uses and constantly controls a tool to produce a desired effect. An example of a ScE is the executing individual using a stick to dig a hole for heat treatment in a sand-bath. The stick is continuously controlled by the individual. In contrast, the SiE refers to an effect that is initiated by the executing individual but not actively controlled in the process. Although the subject can intervene to change, reduce, intensify or stop the effect, at least part of the impact takes place without any active control of the subject. An example of such a SiE is embers transmitting heat to a stone during heat treatment in a fire. (ii) we excluded in all our analyses the effect of the target object on the subject in the standardized last step of every cognigram published so far, known as ‘the satisfaction of need’ (see for example: [16, 31]). We chose to make this exclusion because an effect of a passive focus on an active one contradicts the definition used by Haidle [31, 32] stating that only active foci (i.e. tools) influence or transform other foci.

### Choices made during the interpretation of heating techniques

To interpret the chain of actions associated with the four heating techniques we made a few choices and assumptions. These are: a burning camp-fire exists at the site. We made this theoretical assumption because interpreting the complexity of fire-making lies beyond the scope of this study and there is already published data on fire making [16]. If a heating technique requires a separate fire (e.g. heat treatment in an earth-oven), this fire is lit by picking a burning branch from the camp-fire and lighting wood placed in/on the heating structure. We also assume that if digging a pit is involved in the heating technique, a simple stick is used for it. Such a stick is also used to manipulate embers or the fire itself.

Heat treatment itself is part of a larger modular behaviour, as heat-treated stone was used to produce artefacts that were then (at least potentially) used to achieve a multitude of intended goals. Each possible behaviour that heat treatment was part of consists of three main groups of activities: (I) actions connected to heat treatment itself, (II) activities linked to tool production and (III) activities carried out in conjunction to the use of the produced tool. Therefore, analysing the four different heating techniques as complete sequences of actions requires understanding the processes associated with those activity groups. However, discussing the use of heat-treated stone in a comprehensive manner is beyond the scope of this study. Instead, we focus on the heat treatment procedure itself. Nine activities can theoretically be associated with this procedure: (i) stone acquisition, (ii) acquisition of a stick or several sticks to manipulate the fire or dig a hole, (iii) acquisition of wood-fuel for the fire, (iv) maintenance of the fire / production of sufficient embers and (v) the heat treatment itself. Activities (i) to (iv) are prerequisites for the heat treatment (v). We acknowledge that all these activities are associated with heat treatment and add to the actual procedure (v). However, they are potentially identical in the four heating techniques and thus are irrelevant for the comparison of the complexity between the heating techniques. Thus, the cognigrams associated with steps (i) to (iv) are omitted here. Our comparative analysis is based on the four cognigrams associated with the action of heat-treating itself (v).

## Results

The cognigrams of the four heating techniques are described in order of the one requiring the least foci and steps to the one requiring the most.

### Heat treatment in a fire

**Fig 3:**
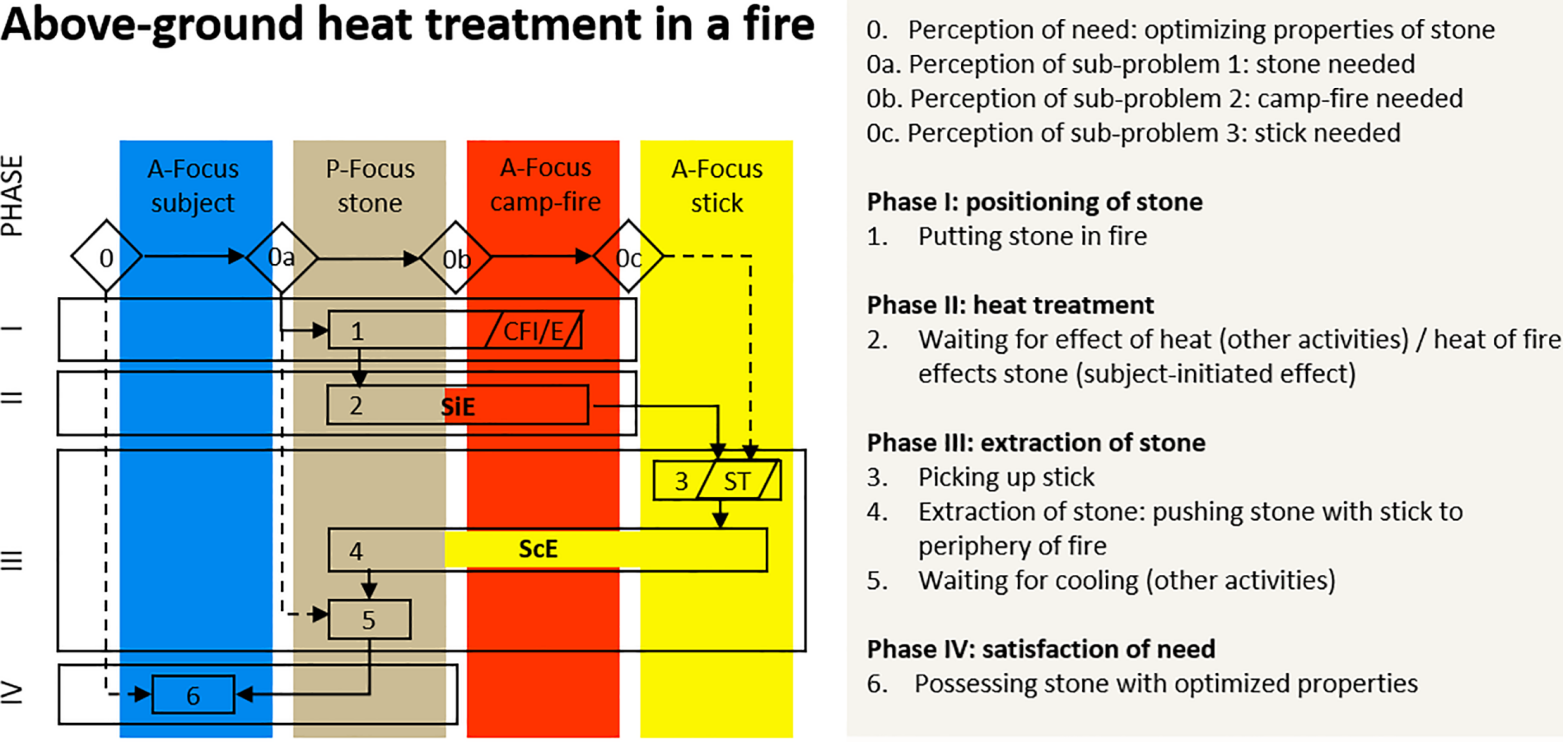
Cognigram of above-ground heat treatment in a fire. The cognigram is based on archaeological evidence and experiments conducted by Schmidt et al. [33].

Fig 3 is the cognigram of above-ground heat treatment in a fire as illustrated in Fig 1A. The first observation stemming from this cognigram is the presence of four foci of attention, implying that the heat treatment instigator must consider three objects in addition to acting itself. Two of them are active foci (the fire and the stick) and one is a passive focus (the stone). The second observation is that the sequence of actions consists of six steps and four phases, suggesting that six different actions must be performed sequentially. The third observation is that on two occasions, a focus has an effect on another focus: one subject-controlled effects and one subject-initiated effects. The ScE is: pushing the heat-treated stone to the periphery of the fire for cooling (phase III, step 4). The SiE is: the heat of the camp-fire affects the stone (phase II, step 2).

### Heat treatment at the periphery of a fire or ‘pile of embers’ technique

**Fig 4:**
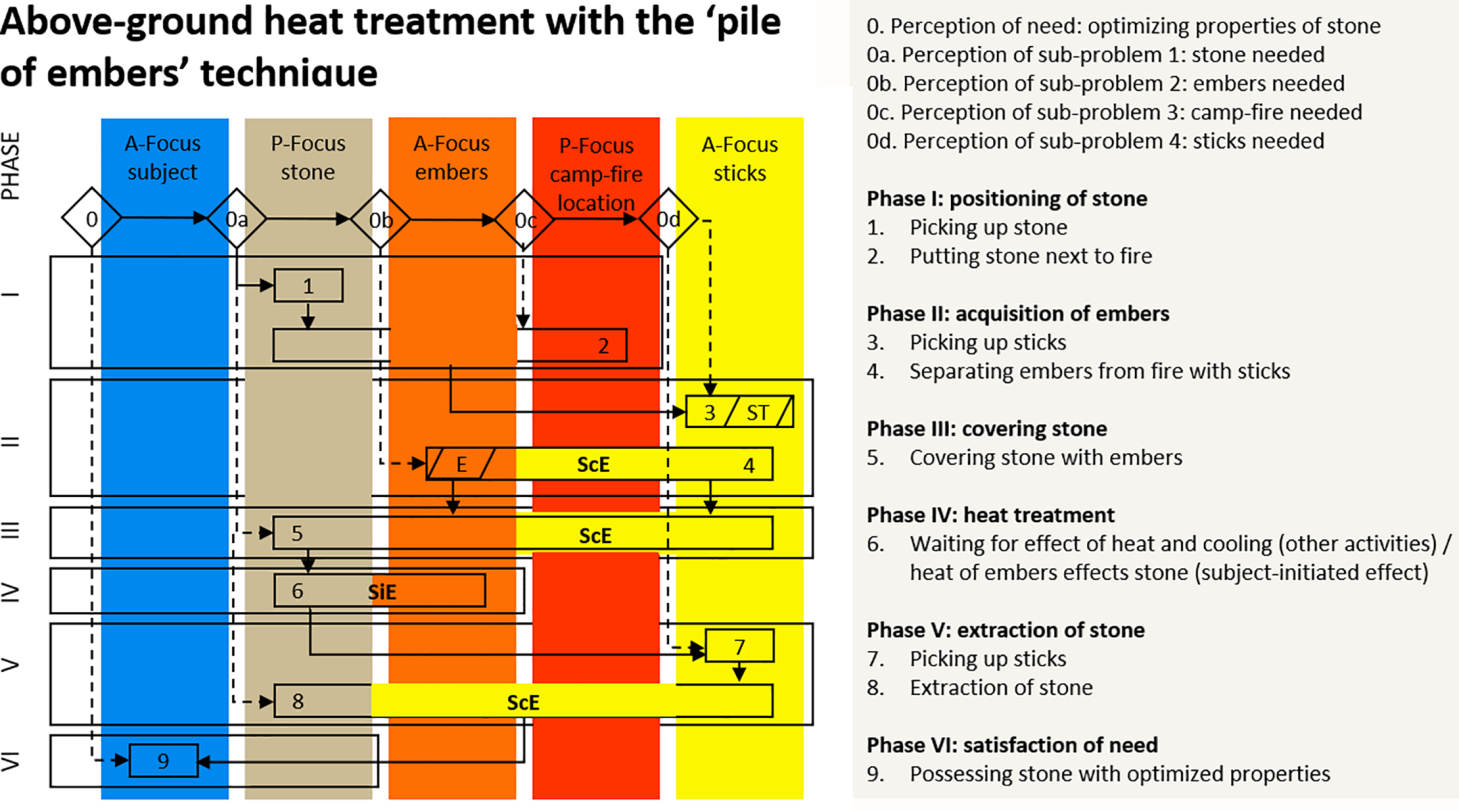
Cognigram of above-ground heat treatment with the ‘pile of embers’ technique‘. The cognigram is based on archaeological evidence and experiments conducted by Schmidt et al. [33].

The cognigram of above-ground heat treatment in a ‘pile of embers’ is shown in Fig 4 (see Fig 1B for illustration of the technique). Three kinds of observations can be drawn from this cognigram. First, the heat treatment instigator has to take into account himself and four additional foci of attention. Two of them (the embers and the sticks) must be controlled in an active way (active foci) and two (the stone and the camp-fire) are passive foci. Second, the sequence of single actions conducted by the heat treatment instigator comprises nine steps and six phases. Third, there are four effects between foci: three times a ScE and one time a SiE. ScEs are: separating embers from the fire (phase II, step 4); covering the stone with embers (phase III, step 5); extracting the heat-treated stone (phase V, step 8). The SiE is: the heat of the embers affects the stone (phase IV, step 6).

### Sand-bath heat treatment

**Fig 5:**
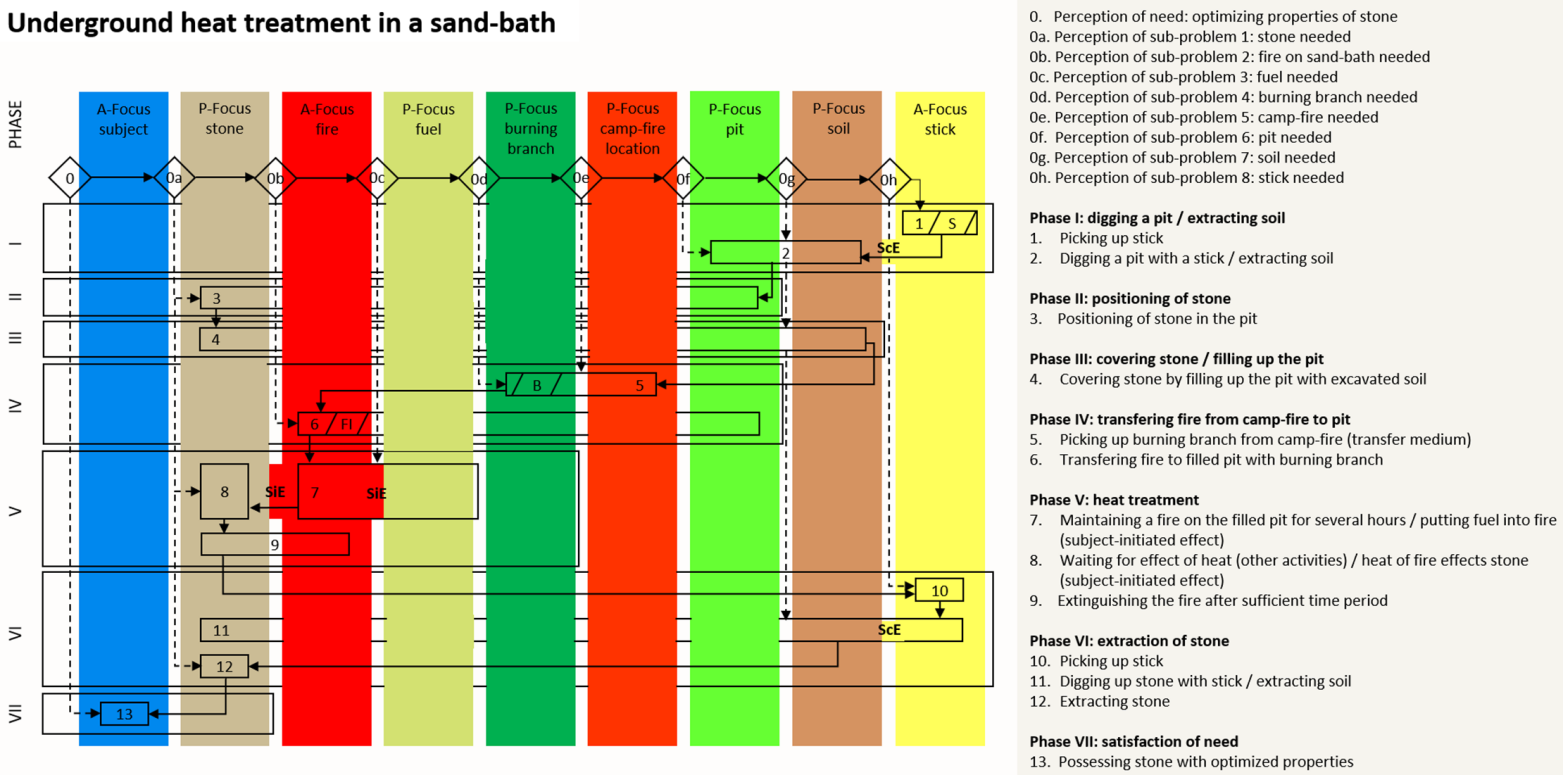
Cognigram of underground heat treatment in a sand-bath. The cognigram is based on archaeological [45] and ethnographic evidence (see for example: [56–58]).

Fig 5 is the cognigram of underground heat treatment in a sand-bath as illustrated in Fig 1C. Eight foci of attention, the instigator himself excluded, are involved in the activity. Two of them are active (the fire and the stick) and six are passive foci (the stone, the fuel, the burning branch, the campfire, the pit and the soil). Thirteen sequential steps and seven phases are necessary for sand-bath heating. Four effects are involved; two being controlled by the instigator (ScE) and two initiated (SiE). ScEs are: digging a pit and extracting the soil (phase I, step 2) and excavating the stones (phase VI, step 11). The SiEs are: the fire burns the fuel (phase V, step 7) and the heat of the fire affects the stone through the sediment (phase V, step 7 and 8).

### Heat treatment in an earth-oven

**Fig 6:**
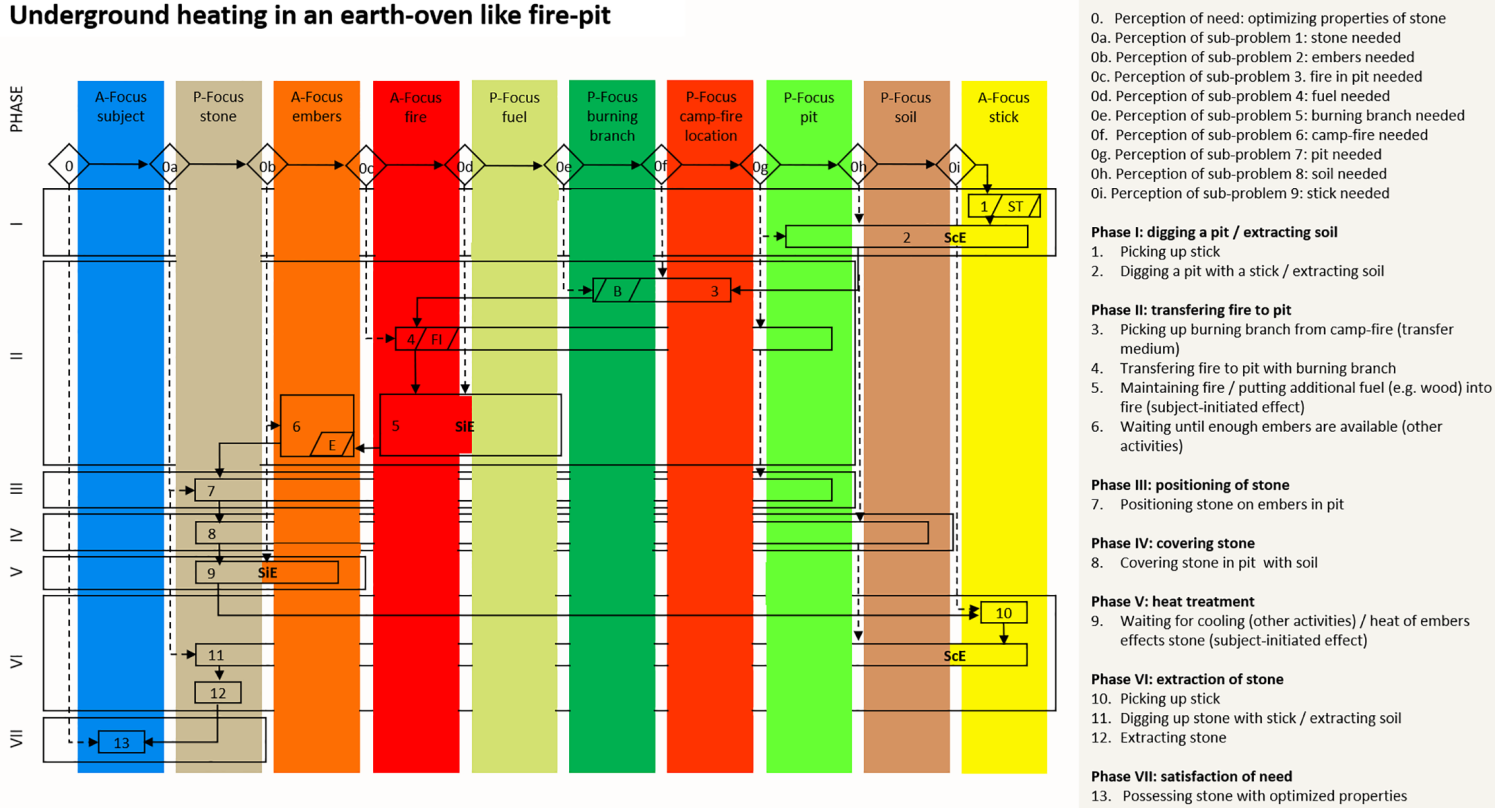
Cognigram of underground heat treatment in earth-oven like fire-pit. The cognigram is based on archaeological evidence [46].

The cognigram of underground heat-treatment in an earth-oven like fire-pit, as illustrated in Fig 1D, is shown in Fig 6. Here the heat treatment instigator has to consider nine foci of attention other than himself. These include three active foci (the embers, the fire and a stick) and six passive foci (the stone, the fuel, the burning branch, the campfire, the pit and the soil). Thirteen sequential steps and seven phases are required to heat-treat stones in an earth-oven. Four effects between foci are involved, two being controlled by the instigator (ScE) and two only being initiated by the instigator (SiE). ScEs are: digging a pit with a stick (phase I, step 2); excavating the heat-treated stone (phase VI, step 11). SiEs are: the fuel is burned by the fire (phase II, step 5); the embers’ heat affects the stone (phase V, step 9).

## Discussion

### Relative complexity of heat treatment techniques

**Fig 7:**
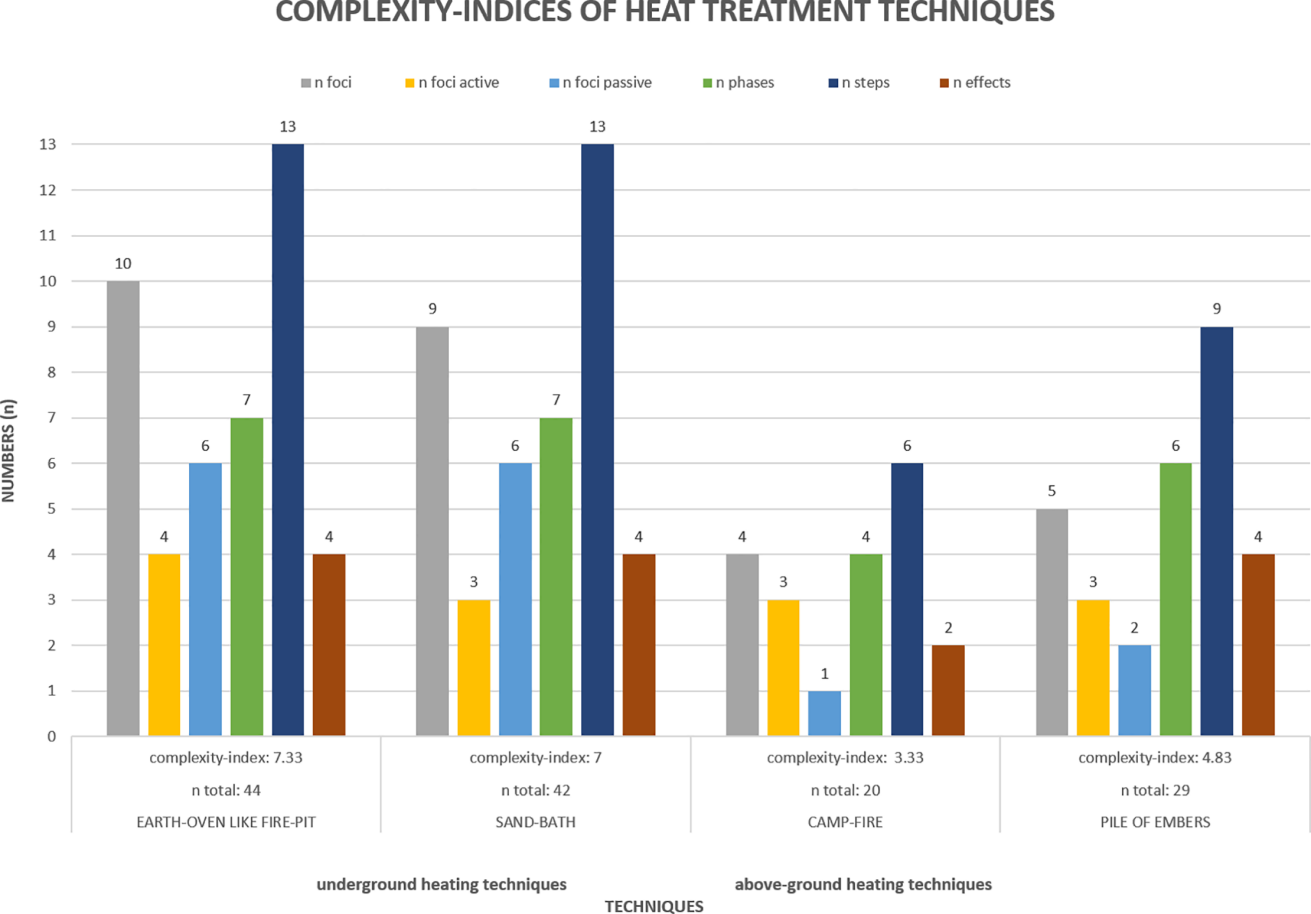
Complexity-indices of heat treatment techniques. Graph of the number of foci (active and passive), phases, steps and effects as well as the total number of elements (n total) and complexity-indices (mean) for the four heating techniques (underground: earth-oven like fire-pit, sand-bath; above-ground: ash-embers-cone and pile of embers). Underground heating techniques show higher complexity indices than above-ground heat treatment. Complexity indices for the heating techniques are: earth-oven like fire-pit: 7.33 (mean value of 10 foci (4 active and 6 passive), 13 steps, 7 phases and 4 effects; sand-bath heating: 7 (mean value of 9 foci (3 active and 6 passive), 13 steps, 7 phases and 4 effects; camp-fire: 3.33 (mean value of 4 foci (3 active and 1 passive), 6 steps, 4 phases and 2 effects; pile of embers: 4.83 (mean value of 5 foci (3 active and 2 passive), 9 steps, 5 phases and 4 effects.

Our analysis of the problem-solution distance associated with these four archaeologically documented heating techniques reveals clear differences in their complexity. The main differences lie in the quantitative measures of complexity that can be assessed through an analysis of the problem-solution distance (i.e. the length and breadth of the operational sequence). By counting the number of items in all four cognigram-categories (active and passive foci; steps; phases; effects) it is possible to quantify part of the techniques’ complexity. The counts per category for all four heating techniques are summarised in Fig 7. We further propose that a complexity index can be derived from these counts if the number of items in each category is averaged. The index is representative of the number of objects necessary, of the number of attention foci that are active and passive, of the length of the sequence of actions, of the number of intermediary goals taken into account (number of phases) and of the effects involved in the process. The index value for heat treatment in a camp-fire is, at 3.33, the lowest of the four procedures. ‘Pile of embers’-heat treatment has an index value of 4.83. Based on these values, heat treatment directly in a camp-fire is less complex than the ‘pile of embers’ technique. This is exemplified by a shorter sequence of actions (six vs. nine steps for heat treatment in a ‘pile of embers’), and the smaller number of tools/objects involved in camp-fire heat treatment (four vs. 5 foci). Also, ‘pile of embers’-heat treatment requires more intermediate goals (six phases vs. four phases for heat treatment in a fire) and more subject-controlled effects (one vs. three for heat treatment in a fire).

Underground heat treatment has a longer problem-solution distance (Fig 7). Both underground heating techniques involve more tools, objects and locations than above-ground heat treatment. The sequence of actions is longer, containing more steps and intermediate goals (phases). These differences are mainly due to digging a pit and lighting a fire in the pit (earth-oven) or on the sediment cover (sand-bath). Neither a pit nor a separate fire are required to heat-treat stone with the above-ground techniques as a regular domestic fire can be used instead. Compared with each other, both underground heat treatment techniques have comparable problem-solution distances. Complexity indices of sand-bath heating and heat treatment in an earth-oven are 7 and 7.33 respectively. Thus, heat-treating stone in an earth-oven is the most complex of the four analysed procedures, followed by sand-bath heating. The two above ground heating techniques are significantly simpler.

Another way of comparing the four heating techniques is by analysing them in terms of qualitative aspects of complexity (i.e. effects and further conceptual cognitive elements). Other than effects (i.e. subject-controlled and subject-initiated effects) no conceptual cognitive elements, like composition, are part of the four heat treatment procedures. We propose that some cognitive parameters can be understood by investigating the nature of effects involved in the techniques. For example, the SiE of placing stone in contact with embers, so that they can transmit their heat to the stone, requires several thought processes: knowing and anticipating the effect of hot embers on other materials, inferring the reaction of stone to heat and estimating approximate reaction kinetics. Although the executing individual can interfere in the process (e.g. by maintaining a fire by putting supplementary wood fuel into it) or completely stop it (e.g. extinguishing the fire), the progression of the process cannot be controlled. This phenomenon must be anticipated beforehand, and the executing individual has to trust in the efficiency of the effect. This is different from a ScE where the individual constantly controls an action. An example of a ScE is stone knapping where all actions are controlled, and unexpected events can be compensated for by the knapper at all times. As with other conceptual cognitive components, like composition, the subject initiated effect opens up an array of new technologies. Anticipating a process throughout its duration and setting it up so that no corrective actions must be undertaken while it is running appear to be key cognitive requirements for the invention of automated processes. These processes demand a more sophisticated planning depth than a process that can be controlled while it is ongoing. In this regard however, all four heating processes are identical because they require the same types of effects: even the simplest of the techniques requires at least one SiE. Thus, regardless of the technique used, heat treatment can be considered a significant MSA innovation that must be understood in terms of its socioeconomic impact for the MSA society.

### Complexity of heat treatment in the framework of Middle Stone Age innovations

**Fig 8:**
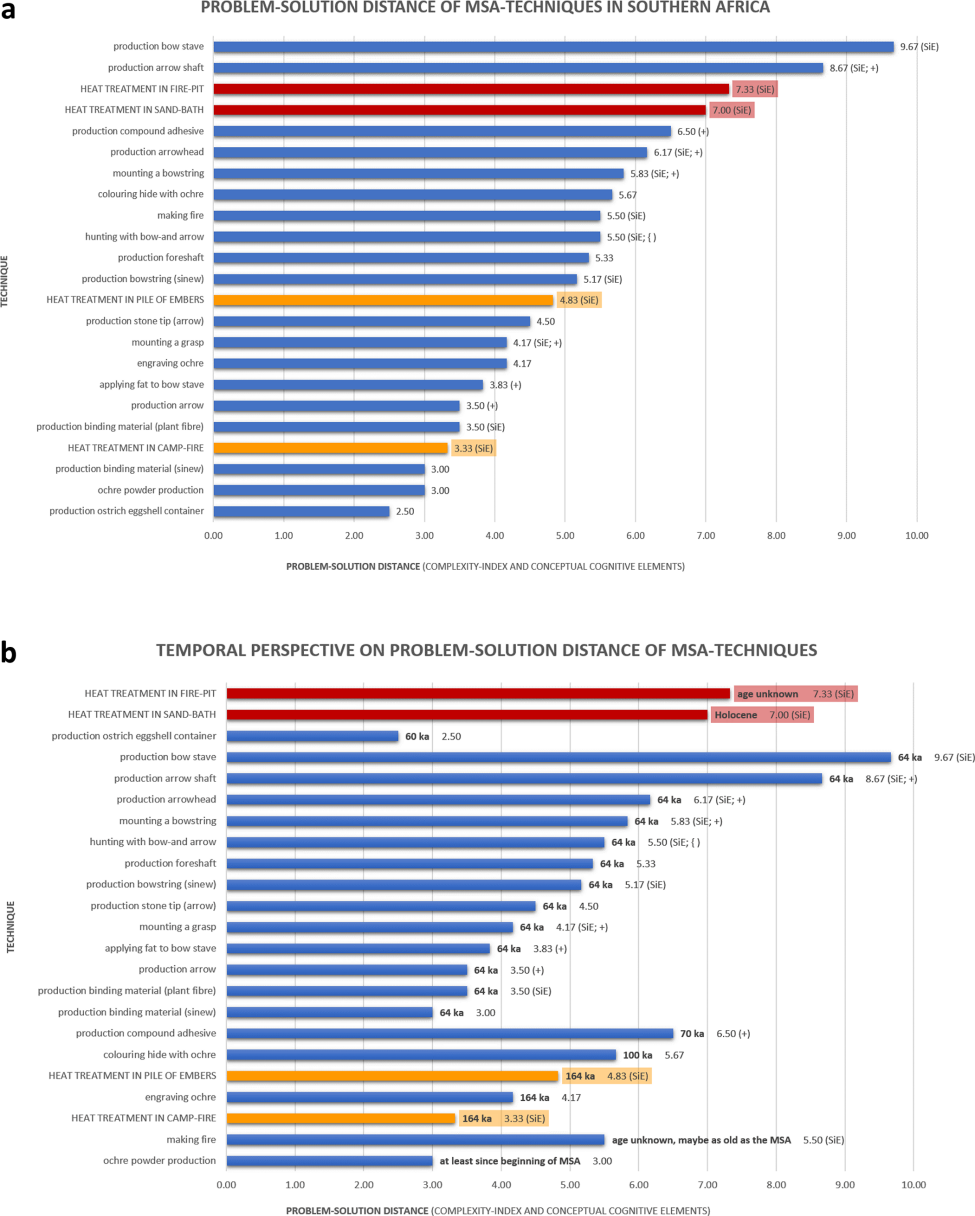
Problem-solution distances (expressed as complexity-index) of 19 Middle Stone Age techniques and the four heat treatment techniques. Techniques involving conceptual cognitive components are labelled as such: subject-initiated effect (SiE), composition (+) and technological symbiosis ({). a: Diagram of the problem-solution distance (complexity-index: average of the number of active foci, passive foci, phases, steps and effects; and conceptual cognitive components) of 19 MSA techniques and the four heating techniques. Heat treatment with the ‘pile of embers’ technique lies roughly in the middle of the 23 techniques. Above-ground heat treatment in a fire is one of the less complex techniques. Underground heat treatment is among the most complex techniques but still in the range of MSA complexity. 13 techniques involve a SiE, 7 techniques composition and one technique technological symbiosis. b: Diagram of the temporal perspective on the complexity of the 19 MSA techniques and the four heat treatment procedures. Nearly all techniques with higher complexity indices than above-ground heating appear later in the current archaeological record of the MSA than above-ground heat treatment or their age is yet unknown. Only fire making predates early silcrete heat treatment. Above-ground heating techniques and probably fire making are the oldest techniques including a SiE (data extracted from [3, 6, 11, 12, 14–16, 45, 46, 64, 66–76]).

Beyond providing data on the relative complexity of these four heating techniques, our study also documents the role of silcrete heat treatment in the broader context of MSA innovations. Can early heat treatment be interpreted as requiring high investment in cognitive effort and strategic thinking as previously proposed [3, 18]? Does it fall within the normal range of technological and behavioural complexity known from other MSA techniques? Does it add to our knowledge of MSA cognition by revealing ‘new’ conceptual cognitive components? To put heat treatment in context, we compare (Fig 8) the cognigrams of our four heating techniques with 19 previously published cognigrams of known MSA processes [16, 64]. These 19 techniques comprise for example the grinding of ochre powder, the colouring of hide with ochre [64], making fire [16], making an ostrich eggshell container [16] and several production techniques involved in making a bow and arrows [16]. To compare their complexity, two levels of analysis appear to be necessary: (i) to compare the quantitative aspects of the problem-solution distance by contrasting complexity indices of the 19 techniques with each other and with the four heating techniques and (ii) to compare the problem-solution distance’s qualitative parts (i.e. the nature of effects and other conceptual cognitive elements) involved in the techniques. The results of comparison (i) are summarised in Fig 8A. Both underground heating techniques lie in the upper segment of the range of complexity index values. Only two processes associated with bow and arrow fabrication are more complex. Underground heating, if it were practised during the MSA, would have been among the most complex techniques for which complexity data has been published, but would still reside within the established range of Middle Stone Age complexity. Yet, the only available archaeological data on the techniques used for MSA silcrete heat treatment [33, 36, 37, 65] do not support underground heating. Instead, early silcrete heat treatment seems to have relied on above-ground heating techniques. The complexity regarding the quantitative aspects of the problem-solution distance of above-ground heat treatment with the ‘pile of embers’ technique (complexity index: 4.83) roughly lies in the middle of the 19 techniques. The heating technique with the lowest index value (3.33), above-ground heat treatment in a camp-fire, is among the least complex techniques, with only the production of binding material (sinew), ochre powder and ostrich eggshell containers showing lower complexity-indices. This indicates that MSA heat treatment falls within the range of normal complexity during this period and could, in its simplest version, even be one of the less complex behaviours in the MSA, keeping in mind that so far only the quantitative aspects of complexity have been considered.

The next step is to provide a temporal perspective to this interpretation by taking into account the earliest documented occurrence of each of the 23 techniques (Fig 8B). Underground heat treatment and techniques associated with five activities have higher complexity indices than the two above-ground heating techniques (i.e. they lie above the over-ground heating techniques in Fig 8A): mixing compound adhesives, colouring hide, engraving ochre, bow and arrow making and making fire.

Heat treatment has been suggested to be as old as 164 ka [17] and, although this first evidence from Pinnacle Point involves only a few artefacts, it is likely that the technique appeared for the first time near the beginning of the second half of the MSA. To date, the only archaeological data available indicate above-ground heat treatment in the MSA. The earliest evidences of mixing compound adhesives date to between ~70 ka and ~65 ka at Sibudu Cave [3, 14, 15]. The earliest indirect evidence of abrading ochre on soft materials, that can be interpreted as colouring hides, comes from use-wear analyses of ~77 ka to ~65 ka old ochre from Sibudu Cave [64] and Pre-Stillbay and Stillbay material (~100 ka and ~75 ka) from Blombos Cave [11, 73, 77]. The oldest engraved ochre piece was found at Pinnacle Point and dates to ~164 ka [6, 72]. Other pieces date between ~100 ka to ~58 ka and come from Sibudu, Klasies River Cave 1 and Blombos [11, 64, 68]. Concerning bow- and arrow-making, several authors [69–71, 78, 79] propose that the technology developed between ~100 ka and ~50 ka in sub-Saharan Africa. Experimental archaeology and use-wear analysis of stone tips may suggest the use of bows and arrows in the ~61 ka to ~64 ka dating Howiesons Poort at Sibudu Cave, Klasies River Cave 2 and Umhlatuzana Rockshelter [69–71, 75, 76]. Thus, mixing compound adhesives, colouring hide and bow and arrow making seem to have appeared later in time than heat treatment. Engraving ochre seems to be either contemporary (at Pinnacle Point) or younger than heat treatment. Concerning the production of fire, it is difficult to detect actual fire-making in the archaeological record because most material evidence left by fires (combustion features and burnt objects) only prove the use of fire and not its production [66, 74]. The earliest evidence of fire use dates to ~1 - ~1.5 Ma and was found at the sites of Swartkrans and Wonderwerk Cave in South Africa and at Koobi Fora FxJj 20 in Kenya [66]. In the MSA fire use becomes common at many sites. The earliest evidence dates to between ~279 ka to ~164 ka at Wonderwerk Cave, Florisbad, Border Cave and Pinnacle Point [66]. Thus, fire use is clearly older than the invention of heat treatment and, although fire making can also be considered part of the whole heating process, it is more complex than the other activities associated with heat treatment.

Silcrete heat treatment is not the most complex technical process known from all the MSA. Nonetheless, if we assume that heat treatment was being performed applying the ‘pile of embers’ technique, it was one of the most complex procedures at the time of its invention. However, if we accept an even simpler procedure, merely putting silcrete blocks into a camp-fire and extracting them after a while with a stick, heat treatment would be among the simplest techniques used in the MSA. These results show that early silcrete heat treatment cannot reasonably be considered a remarkable innovative technique requiring complex behaviour and high planning depth, if we only look at the quantitative aspects of complexity–the length and breadth of the operational sequences.

Therefore, we also have to consider (ii) the effects and other conceptual cognitive elements involved in the 23 analysed techniques to achieve a complete picture of the innovative impact of silcrete heat treatment. Thirteen of these techniques comprise SiEs (see Fig 8A and 8B). If all these techniques are put in temporal context, above-ground heat treatment appears to be the oldest materially documented technique with a SiE. If the early date of 165 ka [17] is accepted for heat treatment, only two of the 23 techniques appeared earlier or roughly at the same time: ochre powder grinding [~100 ka to ~75 ka, 11, 68, 73] and engravings on ochre [~164 ka, 6, 72]. None of these techniques involve a SiE. The 13 techniques that comprise SiEs all appeared later in time than the earliest evidence of heat-treatment of silcrete or their first appearance is not yet unequivocally dated (heat treatment in an earth-oven for example). The date of appearance of fire making is still unknown, but we acknowledge that it must have predated the invention of heat treatment. Thus, heat-treatment alongside making fire seem to be the oldest MSA techniques that required higher planning depth and anticipation. However, broadening our perspective, away from techniques for which published cognigram data are available, and leaving the context of the southern African MSA, it is important to highlight that there also are older techniques that might have involved SiEs. The most prominent techniques are cooking and the production of birch bark tar. There is no direct archaeological data for cooking before the late Middle Pleistocene [80, 81] but cooking was proposed to be an early invention in human evolution, dating back to 2 Ma [82–84]. It appears likely that people cooked food since the moment they regularly used fire (i.e. since the onset of the MSA in Africa and since 400–300 ka in Europe [66, 85]). Although no detailed studies are available so far, it seems safe to admit that at least some potentially used cooking techniques require SiEs: the heat-source’s impact on the food and the cooking time (i.e. reaction kinetics) must be anticipated and cannot be controlled continuously throughout the procedure.

The oldest evidence of birch bark tar comes from the European Middle Paleolithic site of Campitello and dates to ~200 ka (two stone artefacts partly covered in tar) [86]. Other sites dating to ~120–80 ka, such as Inden-Altdorf [87, 88] and Königsaue [89, 90], also yielded evidence of birch bark tar. The procedure used for birch tar production is still unknown [91] and no comment on the potential implication of SiEs can be made here. Thus, at least cooking is older than heat treatment and involves a SiE. MSA silcrete heat treatment can therefore not be viewed as an unpreceded innovation.

## Conclusion

Our study highlights the importance of silcrete heat treatment in the southern African MSA by showing that it was one of the most complex techniques at the time it was invented. Previous authors were correct in interpreting heat treatment as an outstanding innovation. At the time it was discovered in the MSA, possibly as early as 165 ka, heat treatment required higher planning depth and a higher degree of anticipation than many other contemporaneous techniques. At least as early as this date, people were able to anticipate the effect of heat on stone and to estimate approximate reaction kinetics. This ability opened up the possibility for new techniques that involve subject-initiated effects and may represent the onset of other complex automated processes in the MSA. Only from about 100 ka on, heat treatment falls within the normal range of complexity of commonly used techniques.

It must nonetheless be stressed that, outside of the context of the southern African MSA, there were older techniques that required understanding and anticipating of effects: cooking and the production of birch bark tar require a similar skill-set and are either older than (cooking), or roughly contemporaneous (birch tar) to heat treatment. It is thus likely that the cognitive bases for high planning depth are substantially older than the MSA and the Middle Palaeolithic, maybe even as old as 2 Ma. An early origin of these cognitive capacities in human evolution may also explain that both European Neanderthals and African anatomically modern humans invented techniques that involve effect anticipation and automated steps. The exact role of initiated effects in commonly used Neanderthal techniques has yet to be analysed and future studies should shed light on the similarities and differences between early cultural expressions on different continents.
